# Extracellular matrix production *in vitro* in cartilage tissue engineering

**DOI:** 10.1186/1479-5876-12-88

**Published:** 2014-04-05

**Authors:** Jie-Lin Chen, Li Duan, Weimin Zhu, Jianyi Xiong, Daping Wang

**Affiliations:** 1 Shenzhen Key Laboratory of Tissue Engineering, Shenzhen Second People’s Hospital (The First Affiliated Hospital of Shenzhen University), Shenzhen 518035, Guangdong Province, China; 2 School of Medicine, Sun Yat-sen University, Guangzhou 510182, Guangdong Province, China; 3 Department of Orthopedics, Shenzhen Second People’s Hospital, Shenzhen 518035, Guangdong Province, China

**Keywords:** Cartilage, Tissue engineering, Extracellular matrix, Collagen type II

## Abstract

Cartilage tissue engineering is arising as a technique for the repair of cartilage lesions in clinical applications. However, fibrocartilage formation weakened the mechanical functions of the articular, which compromises the clinical outcomes. Due to the low proliferation ability, dedifferentiation property and low production of cartilage-specific extracellular matrix (ECM) of the chondrocytes, the cartilage synthesis *in vitro* has been one of the major limitations for obtaining high-quality engineered cartilage constructs. This review discusses cells, biomaterial scaffolds and stimulating factors that can facilitate the cartilage-specific ECM production and accumulation in the *in vitro* culture system. Special emphasis has been put on the factors that affect the production of ECM macromolecules such as collagen type II and proteoglycans in the review, aiming at providing new strategies to improve the quality of tissue-engineered cartilage.

## Introduction

Articular cartilage lesions, once caused by trauma or pathology, are hard to heal by itself due to the poor capability of chondrocytes to regenerate. The treatment for articular cartilage lesions is versatile and challenging, which develops from initial microfracture, mosaicplsty to the first (periosteum as the cover for the defects) and second (collagen type I/III as the cover for the defects) generation of autologous chondrocyte implantation (ACI) and gradually widely used matrix-assisted autologous chondrocyte implantation (MACI) [1,2]. The outcome of the treatment improves with the development of the techniques derived from cartilage tissue engineering. However, there are always some cases with fibrocartilage repair instead of hyaline cartilage regeneration, resulting in inferior mechanical functions of the cartilage [3].

Cartilage is a special avascular and aneural tissue with sparse chondrocytes embedding in the dense extracellular matrix (ECM) and the cartilage ECM macromolecules play a central role in cartilage functionality, primarily in the establishment of the mechanical functions [4]. Collagen type II, proteoglycan and glycosaminoglycan (GAG), as the primary compositions in the ECM, have always been used as the criteria for the identification and evaluation of the chondrogenic capacity of cells and constructs obtained for cartilage tissue engineering. Parameters such as GAG content, cell mobility and water content usually can be reached up to the level of native tissue. However, the quantity of collagen type II (the major molecule for cartilage mechanical functions) is much lower from cells *in vitro* than that from the native cartilage [5,6]. Therefore, an obvious obstacle currently is the inadequate production and accumulation of some chondrocyte-specific ECM macromolecules, especially collagen type II in cultured constructs comparing with native cartilage tissue, even though there has been a great progression in regeneration of the cartilage lesions using engineered constructs [5]. As collagen type II to a large extent provides the tissue with mechanical functions, strategies to promote its production and accumulation in engineered tissues need to be stressed.

This review integrates cells, biomaterial scaffolds and stimuli that can facilitate ECM production and accumulation in cartilage tissue engineering (Table 1), with special focus on their effects on collagen type II expression and production. We endeavor to cover the above aspects that can improve the quality and function of engineered constructs, in the hope of promoting new technology to produce high-quality tissue engineered cartilage.

**Table 1:** Summary of cells, scaffolds and stimuli

**Cells**	Cell sources	Chondrocyte	[6,7,12,13,15]
		Stem cells	[8-11,14,15]
		High chondrogenic cells	[16-18]
	Cell culture	Medium	[13,17,22-24]
		Coating surfaces	[25-29]
		Co-culture	[30,32-34]
**Scaffolds**	Collagens	Type I/III Collagen, type II collagen	[37-40]
	Composite scaffolds	PGA/fibrin, PLGA/PLLA, PLGA/collagen, peptide/gene modified scaffolds	[41,42,44-50]
	Novel scaffolds	Nanofiber, Silicone rubber dish	[51,52]
	ECM-based scaffolds	Cartilage/cell-derived ECM scaffolds	[54-56]
**Stimuli**	Growth factors	TGFβs, BMP2, IGF1, CCN2	[38,58-64]
	Compounds	Hydrocortisone, icariin, avocado	[19,67-70]
	Gene therapy	BMPs, IGF1, TGFβs, miRNA, shRNA	[20,21,38,65,72-76]
	Environmental factors	Hypoxia, pressure, bioreactor, cryopreservation	[30,64,78-81,83-85]

### Cells

#### Cell sources

The cell sources employed in cartilage tissue engineering range from chondrocytes, stem cells to gene-modified cells. Several comparative studies revealed that chondrocytes cultured *in vitro* could produce more collagen type II than that of its stem cell counterparts as the collagen type II concentration was much higher in cultured chondrocytes [7,8]. Shahin *et al.* used human chondrocytes for constructing tissue-engineered cartilage and the obtained collage type II concentration was around 8.5% of dry weight tissue. In contrast, in a similar study using human adipose-derived stem cells, the concentration of collagen type II was only 0.22% of dry weight tissue. However, adipose-derived stem cells showed inferior properties in chondrogenesis comparing with synovium-, bone marrow- and periosteum-derived stem cells in both human and rat [9,10]. Synovium-derived stem cells have been proved to be superior for the production of cartilage matrix including collagen type II and have the greatest ability for chondrogenesis, among the other stem cell sources [1]. As for the comparison between chondrocytes and synovium-derived stem cells, there was a research demonstrating synovium-derived stem cells and BMSCs produced more GAGs and collagen than chondrocytes [11]. However, the chondrocytes used in the research were P2 cells in monolayer culture (primary culture was termed P0 here), of which the collagen type II and aggrecan significantly decreased as shown in other studies and also in our experiments using a rabbit model (Additional file 1, Figure 1) [12,13]. Therefore, more investigations need to be explored for the verification. Nevertheless, a recent study showed that fibrocartilage-like tissue produced from synovium-derived stem cells was found in the superficial layer of cultured constructs [14]. While in another study comparing human bone marrow stromal cells (HBMSC) and articular chondrocytes, cartilage-like tissue derived from chondrocytes was found in both superficial zone-like and middle zone-like structure. By contrast, relatively thicker fibrous capsules were shown in constructs from HBMSCs [15]. As superficial zone of the cartilage bears maximum load, it needs to be paid special attention to in cartilage tissue engineering. Chondrocyte and stem cells mentioned above have been summarized in Table 2.

**Figure 1:**
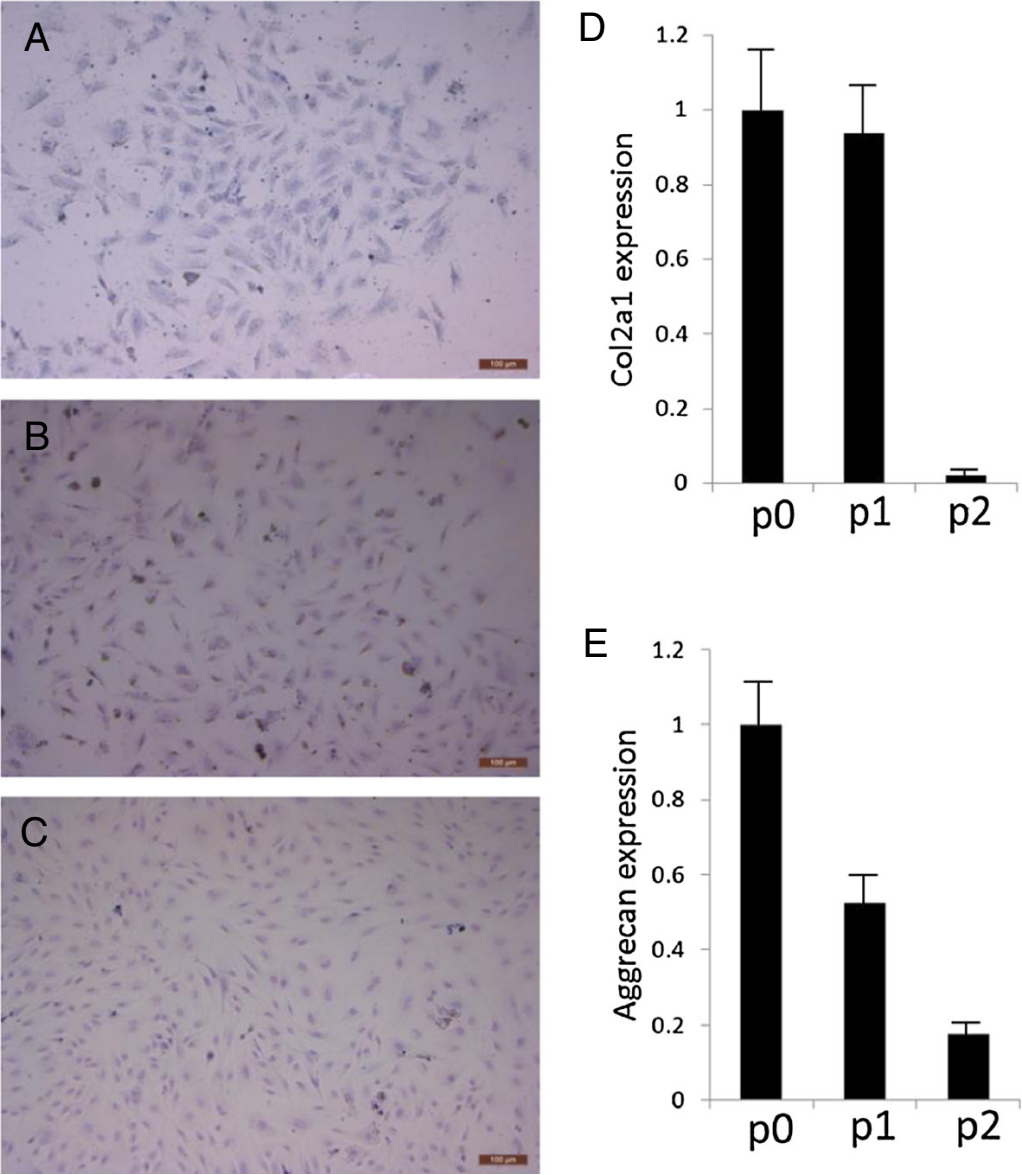
**Evaluation of GAGs, Col2a1 and aggrecan in passaged rabbit chondrocytes. (A-C)** Toluidine blue staining showed production of GAGs from cells declined following passaging from P0 **(A)**, P1 **(B)** to P2 **(C)**. Scale bar: 100 μm. **(D-E)** Real time PCR showed the expression of Col2a1 **(D)** and aggrecan **(E)** decreased with cell passaging.

**Table 2:** Summary of chondrocyte and stem cells in the part of cells

**Cell type**	**Species**	**Passage**	**Culture method**	**Reference**
Chondrocyte	Human	P2	Monolayer + 3D	[7]
ADSC	Human	P4	Monolayer + 3D	[8]
SDSC	Human	P2	Monolayer + 3D	[11]
BMSC	Human	P2	Monolayer + 3D	[11]
Chondrocyte	Human	P2	Monolayer	[11]
Chondrocyte	Rabbit	P0-P4	Monolayer	[12]
Chondrocyte	Human	P0-P4	Monolayer	[13]
SDSC	Human	P4-P7	Monolayer + 3D	[14]
Chondrocyte	Human	P0-P4	Monolayer + 3D	[15]
BMSC	Human	P1-P4	Monolayer + 3D	[15]

ADSC: adipose-derived stem cell; SDSC: synovium-derived stem cell; BMSC: bone-marrow derived stem cell; Monolayer + 3D: monolayer culture followed by 3D culture.

Sorted single cell clone and clonal cell strains were also investigated for chondrogenic capacity. Fluorescence sorted chondrocytes with brighter CD49c and CD44 were detected to expression more collagen type II and higher levels of GAG than unsorted cells [16]. Magnetically sorted stromal cells (STRO-1) from human bone marrow mononuclear populations have been used as seed cells with alginate-chitosan encapsulating and were proved to undergo chondrogenesis with increased collagen type II and proteoglycan in micromass culture and also in culture using a perfused bioreactor [17]. Induced pluripotent stem cells (iPSCs) were also used for screening cells with high chondrogenic capacity. Fluorescence sorted mouse fibroblast-derived iPSCs with GFP driven by Col2 promoter/enhancer showed higher levels of collagen type II, aggrecan and GAGs [18]. Therefore, identification of chondrocyte-specific markers and sorting of cells with relatively higher chondrogenic capacity would provide a new means for the seeding cells for cartilage tissue engineering.

Gene-modified cells employed in cartilage tissue engineering have been paid more attention to nowadays with the rapid development of molecular biology and technology, including cells with single gene transduction and co-transduction of two genes or a gene with an shRNA [19-21]. All these methods aim at improving the chondrogenic capacity of cells and the quality of engineered constructs. As for the details of genes and gene-modified cells that can promote ECM synthesis and collagen type II expression, they will be elucidated in the later part of *gene therapy*.

#### Cell culture

Dedifferentiation is the major cause of degeneration of chondrocytes *in vitro* and maintaining chondrocytes phenotype and features is always on focus. Likewise, for stem cells, chondrogenesis is the primary process to chondrocytes and differentiation of stem cells has been broadly investigated. Optimization of culture methods improves characteristics of chondrocytes and facilitates collagen type II and proteoglycan production *in vitro*. There are various ways for chondrocytes culture and chondrogenesis of stem cells, including using different media, coating proteins and co-culture systems. Different scaffolds and growth factors are also involved, which will be discussed later.

A study specifically designed to optimize the chondrogenic medium using factorial design of experiments suggested standard chondrogenic medium with increased level of TGF-β1 and glucose and decreased level of dexamethasone could promote collagen type II to collagen type I ratio (ColII/ColI) and better maintain the chondrocyte properties [22]. TGF-β1 in serum free medium has been proved to increase collagen type II expression and better maintain human chondrocyte phenotype comparing with medium plus fetal calf serum (FCS) [23]. Moreover, the redifferentiation of serum free medium cultured monolayer chondrocytes in a 3D scaffold was observed with the expression of collagen type II and proteoglycans; while it was not observed for chondrocytes and STRO-1 cells cultured in FCS supplemented medium [17,23,24]. One recent study showed chondrocytes cultured in medium supplemented with 20% platelet-rich plasma produced thicker cartilage tissue than cells in medium with 20% FBS [25], suggesting platelet-rich plasma was more able to maintain chondrocyte properties *in vitro*.

Different coatings of cell culture surfaces influences chondrocyte culture *in vitro*. Cells cultured on collagen type I and II coating dishes expressed more collagen type II expression and produced more glycosaminoglycan (GAG) than uncoated control samples [26,27]. Though not significantly, higher collagen type II expression was observed in chondrocytes on collagen type II coated dishes than collagen type I coated dishes. On the contrary, fibronectin was suggested to promote collagen type II degradation [28]. More specific research came from Shawn *et al.,* who investigated the effects of coating with zone-specific cartilage extracellular matrix molecules on cartilage formation [29]. Cells cultured in monolayer showed different outcomes with pellets culture. While hyaluronic acid (HA) and osteopontin coating increased Col2a1expression in monolayer culture, pellets culture of cells harvested from HA coated dishes showed decreased expression of collagen type II. On the contrary, tenascin C promoted collagen type II expression in pellet culture while inhibited its expression in monolayer culture. Cells on type II collagen coated dishes showed consistent increased expression of Col2a1 with both monolayer culture and pellet culture system [29]. As different coatings and different culture environments synergistically affect the cell behaviors, both need to be taken into considerations in an experimental design. Aside from protein coating, TiO2-coated coverslips were used for human MSCs culture and proved to significantly enhance cell proliferation without weakening the chondrogenic capacity [30], which would also benefit the ECM production.

Cell co-culture systems have been used in many fields of biomedical sciences. As for cartilage tissue engineering, co-culture of MSCs and chondrocytes provide us with a means to obtain promising cells. After a comparative study of chondrocytes, MSCs and co-culture of chondrocytes and MSCs, the co-cultured cells were suggested to be the most prospective for cartilage tissue engineering [31]. Co-culture of primary chondrocytes with MSCs or embryonic stem cells or iPSCs increased collagen type II and Sox 9 expression and promoted cartilage matrix formation [3,32,33]. The optimal ratio of MSCs and chondrocytes in co-cultures for cartilage engineering varied in different studies. Some study revealed that higher ratios of human MSCs to human articular chondrocytes was more beneficial to chondrogenesis as was shown by newly synthesized cartilaginous ECM and collagen type II gene up-regulation [34]. However, another study suggested 63:1 as the appropriate initial ratio of MSCs/chondrocytes for enough stable chondrocyte-like cells and the constructs from co-cultures were more cartilaginous than that from chondrocytes [35].

Development and optimization of cell culture methods aiming at elevating the chondrogenic capacity of seed cells will improve the cartilage-specific ECM production and finally benefit the cartilage tissue engineering with relatively higher quality of engineered constructs.

### Scaffolds

There are various scaffolds used for cartilage tissue engineering as a 3D environment facilitates to maintain chondrocyte properties than monolayer culture. The characteristics of a scaffold involve mechanical strength, biocompatibility, biodegradability, porosity and toxicity [36]. The scaffold must be structurally stable, allow cells to infiltrate and growth factors to attach. All these characteristics of scaffolds provide cells with healthy microenvironment, beneficial cell-cell interaction and adequate nutrient exchange *in vitro*. Thus, the constructs with cells and ECM mimic the cartilage and are more close to the native cartilage tissue than monolayer cells when implanted in the defects. Of so many scaffolds used, we will focus on clinically used scaffolds and those assist to promote ECM production, especially collagen type II production here.

Collagen is a major component in the cartilage matrix and collagen scaffold has long been used for cartilage repair [37]. Bilayer type I/III collagen membrane has been clinically used worldwide for ACI and MACI and collagen type II production and regeneration of hyaline-like cartilage were obtained during the observations [38-40]. Collagen type II scaffold was also investigated for cartilage repair and *in vivo* study revealed newly formed cartilage structure the same with normal cartilage [41]. Fibrin glue was often limited to use due to its shrinking feature *in vivo*. However, when it combined with polyurethane, this new composite scaffold improved cell survival ability and increased the expression of collagen type II and also aggrecan [42]. Polyglycolic acid/fibrin (PGA/fibrin) scaffolds used for chondrocyte culture also enhanced the redifferentiation of ovine chondrocytes and the induction of collagen type II and aggrecan after culture. Biomechanical tests further supported this scaffolds for chondrocyte culture with a high tensile strength of 3.6 N/mm2 [43]. Poly(L-lactic acid) (PLLA) and poly(lactic-co-glycolic) acid (PLGA), as materials that have been approved by the US Food and Drug Administration for human clinical uses [44], have been often used for the research of cartilage tissue engineering. Comparing with PLLA, collagen type II and Arg-Gly-Asp (RGD) peptide modified PLLA/PLGA (50:50), collagen type II modified PLLA/PLGA (50:50) exhibits higher collagen type II expression and shows superiority for chondrogenesis both *in vitro* and *in vivo*[45]. Besides, based on type I collagen-PLGA scaffold, improved funnel-like collagen-PLGA hybrid scaffold was shown to be a stronger promoter for cartilage regeneration [46]. PLGA scaffold with hyaluronic acid incorporated substantially promoted collagen synthesis [47]. Moreover, as PLGA microspheres have been successfully used to immobilize DNA, siRNA and growth factors [48,49]. Sox9 expression plasmid loaded PLGA microspheres have been investigated for chondrogenesis. Human MSCs seeded on Sox9 gene and heparinized TGF-β3 coated dexamethsone loaded PLGA microspheres could drastically increase collagen type II expression by 30 times compared with control [50]. Therefore, gene incorporation provides a new and promising strategy to optimize the property of scaffolds for tissue engineering.

Comparing with the traditional scaffolds, more and more novel scaffolds have been under investigations, including peptide-modified scaffolds and mobile scaffolds. Mesenchymal stem cells cultured in Arg-Gly-Asp-Ser (RGDS) peptide incorporated PEG-based hydrogels had greater gene expression of collagen type II and produced significantly more GAGs comparing with control [51]. A newly designed synthetic link N peptide nanofiber was suggested to remarkably promote collagen type II and aggrecan production [52]. Since cell expansion would lead to contact inhibition and to avoid the dedifferentiation of chondrocytes through passaging, mobile scaffold was also examined for chondrocyte culture. Differently from traditional biomaterial scaffolds, the culture surface area of the high-extension silicone rubber dish could increase by 8 folds with a motorized device [53]. Thus, adequate cells could be obtained and extracellular matrix was simultaneously maintained, including collagen type II, which would drastically decrease through passaging.

ECM-based scaffold is an emerging approach in the field of cartilage tissue engineering in that it may be relatively easy to retain the natural growth factors in the ECM [54]. Cartilage-derived and cell-derived ECM scaffolds have been investigated and cartilage-like tissues have been observed [55-57]. However, the quality of formed cartilaginous tissue needs to be evaluated and compared. With improvement of the ECM-based scaffold, it would probably be a promising type of scaffolds for cartilage tissue engineering.

### Stimuli

#### Growth factors and compounds

Various growth factors have been used for cartilage tissue engineering. Members in TGF-β family have been widely used to induce the chondrogenesis of MSCs and enhance ECM synthesis. TGF-β3 was proved to be more capable to induce chondrogenesis of MSCs and also increase the expression of collagen type II and aggrecan [41,58]. As for collagen type II production, the effect of TGF-β1 used in different culture systems was controversial. Depending on the design of the experiments, TGF-β1 could either increase or decrease the expression of collagen type II [59-61]. BMP-2 and IGF-1 both impact chondrogenesis and promote collagen type II production. In a study which compared the effects of BMP-2, IGF-1 and their combination, BMP-2 treatment showed superior ability for collagen type II expression than IGF-1 [39]. Synergistical use of BMP-2 and BMP-7 gave better outcome of ECM production [62]. A more recent study showed standard chondrogenic medium plus TGF-β3, BMP-2 and IGF-1 demonstrated stronger capability for MSCs to produce collagen type II comparing with the other three groups including standard medium with TGF-β3 alone, TGF-β3 + BMP-2 and TGF-β3 + IGF-1 [63]. Another growth factor, connective tissue growth factor (CCN2) has also been proved to strongly enhance the cartilaginous ECM production, including collagen type II and was suggested to reduce age-related changes of articular cartilage [64-67].

Besides the growth factors, some compounds are also proved to effectively induce the expression of collagen type II and other ECM proteins. Hydrocortisone has been found to increase the ability of human articular chondrocytes to produce ECM macromolecules and icariin was proved to help the ECM synthesis in rabbit articular chondrocytes [68-70]. Avocado/soybean unsaponifiables was also identified to facilitate aggrecan and collage type II expression in an osteoblast/chondrocyte co-culture system [19]. Additionally, yeast hydrolysate had been verified to be able to stimulate collagen type II expression and inhibit MMP-13 production in chondrocytes, protecting the cartilage from degradation [71]. However, as for the mechanisms and their clinical use in cartilage tissue engineering, further investigations need to be performed.

#### Gene therapy

Gene therapy has been grown rapidly and is widely used in biomedical sciences. Although gene therapy is in its infancy for cartilage engineering, it shows promising to contribute to the cartilage repair. Often, growth factors are been transfected into chondrocytes or MSCs to improve the properties of cells. IGF-1 and TGF-β1 have been successfully transfected into articular chondrocyte and BMSCs separately and both were proved to increase the expression of collagen type II and aggrecan [20,72]. Meanwhile, functional study further demonstrated IGF-I transfected implants accelerated the regeneration compared with lacZ implants [20]. Furthermore, one recent study investigating co-transduction of different gene combinations which involved any two of genes IGF-1, FGF-2, TGF-β1 and SOX-9 with adenovirus delivering system showed that cells co-transduced with IGF-1 and FGF-2 could significantly express higher aggrecan, collagen type II and other ECM proteins than other combinations [21].

In addition to delivering growth factors to cells to increase cartilage-specific gene expression, down regulation of some negative factors has also been proved to effectively promote collagen type II and aggrecan expression. MicroRNA-181b (miR-181b) has been identified to be a negative modulator for cartilage development and siRNA targeting miR-181b induced collagen type II expression and significantly reduced degeneration of cartilage [73]. MiR-145 was proved to target Sox9 and suppress the chondrogenesis of mesenchymal stem cells; while anti-miR-145 inhibitor could rescue the expression of Col2a1, aggrecan and relevant chondrogenic marker genes [74]. Besides, down regulation of some pro-inflammatory factors that could induce cartilage destruction, such as IL-1, IL-6, TNF-α, would also help cartilage regeneration [68,75,76]. Therefore, inhibition of the negative modulator could be a promising therapeutic strategy for cartilage regeneration. Furthermore, targeting chondrocyte COL1A1 with siRNA could significantly improve chondrocyte phenotype, with increased ratio of COL2A1/COL1A1 and improved cartilage-like matrix formation [39].

Furthermore, the combination of delivering growth factors and down-regulation of negative modulators has also been investigated recently. Co-transduction of TGF-β3 with lentiviral transduction system and shRNA targeting Col1a1 with adenoviral vectors demonstrated optimal efficacy for collagen type II expression and cartilage formation [77]. As gene therapy is just arising in cartilage tissue engineering, much needs to be explored in this field.

#### Environmental factors

As cartilage resides in a microenvironment with reduced oxygen and various biomechanical stresses, creating a similar environment *in vitro* may provide a way to maintain the characteristics of chondrocytes. Hypoxia with 5% or even 3% oxygen stimulated collagen type II expression and biosynthesis of hyaline-like cartilage matrix [39,78,79]. For dynamic environment, expression of collagen type II and aggrecan were induced after the stimulation of a uniaxial compressive load with 1kPa, 1Hz for 30 min [80]. Intermittent hydrostatic pressure benefit long-term chondrocyte culture and the cartilaginous construct formation [81]. As for tensile strength, abnormal cyclic tensile strain inhibited collagen type II expression; while proper and cyclic tensile strain would promote collagen type II expression [65,82]. To provide an environment that could enhance nutrient transport and maintain dynamic state for cells, bioreactors have been used for cartilage tissue engineering. Combined with scaffolds as the support for cells, the system facilitates chondrocyte proliferation and ECM accumulation comparing with static culture [83,84]. Improved bioreactor as wavy-wall bioreactor better increased the cartilage matrix than common dynamic culture using spinner flasks [84]. Perfusion bioreactor was also used to investigate chondrogenesis and chondrocyte growth. It was found that human articular chondrocytes within alginate beads only produced collage type II in a perfused environment. Collage type II was not detected in the static culture condition by contrast [17]. However, one problem caused by dynamic loading was less accumulation of matrix in the scaffold as part of the newly synthesized molecules would disperse into the medium [85]. Besides dynamic environment mentioned above, cryopreservation has a negative effect on the chondrogenic capacity of chondrocytes. Human chondrocytes showed significantly decreased collagen type II expression after cryopreservation [86]. Although new methods and scaffolds are being under investigation for the preservation and revitalization of articular cartilage or chondrocytes [87-89], it is better to use freshly cultured chondrocytes for clinical application if it is possible.

## Conclusions

Cartilage tissue engineering mainly consists of seed cells, scaffolds, stimuli and environmental factors. As cartilage is more specific than other tissues with sparse chondrocytes and dense supportive ECM, emphasis should be put on cell proliferation, differentiation and cartilage-specific ECM production. Low proliferation ability and dedifferentiation property of the chondrocytes limit fine cartilaginous construct formation and use, as well as the efficacy of current therapies. Through the selection of cell sources, optimization of cell culture methods and rise of innovative biomaterial scaffolds, tissue engineered constructs with enhanced cartilage-specific ECM production are promising in the near future. New technologies employing comprehensive factors may make the tissue-engineered cartilage successful and lead the clinical approaches from ACI, MACI to the ideal tissue-engineered cartilage implantation (TEC).

## Competing interest

The authors declare that they have no competing interests.

## Authors’ contributions

CJL conceived of the study, participated in the experiments and drafted the manuscript; DL participated in the design of the draft and revised it critically for important intellectual content; ZW participated in the experiments and interpreted the data obtained; XJ critically revised the draft for important intellectual content and obtained funding; WD supervised the study, revised the draft for important intellectual content, obtained funding and approval of the final version of the manuscript. All authors read and approved the final manuscript.

## Supplementary Material

Additional file 1 Culture and characterization of rabbit chondrocyte. Click here for file
