# Supplementary material for: Extracellular matrix production in vitro in cartilage tissue engineering
Source: J Transl Med. 2014 Apr 5;12:88. doi: 10.1186/1479-5876-12-88 (PMC4233628; doi:10.1186/1479-5876-12-88)
Supplement: Additional file 1 — Culture and characterization of rabbit chondrocyte. [file 1479-5876-12-88-S1.docx]

Supplementary information

**Materials and methods**

*Rabbit chondrocyte culture*

Two-month-old New Zealand white rabbits were provided by the Medical Laboratory Animal Center of the Guangdong Province for the study. All the animal handling procedures were approved by the Ethics Committee of Shenzhen Second People’s Hospital. The rabbits were anesthetized to death and the cartilages from the knees were cut and rinsed with PBS plus penicillin and streptomycin (Gibco) for 3 times. The cartilages were then cut to about 1 cubic millimeter and digested with 1mg/ml collagenase II (Sigma) at 37 ℃ for 5 h with shaking. Cells were collected after filtration and centrifugation and resuspended with DMEM/F12 (Thermo Scientific Hyclone) plus 10% FBS (Gibco) and non-essential amino acid (Sigma). The cells, termed as P0, were seeded at 4× 10^3^ cell/cm^2^ in 6-well plates. Media were refreshed every 3 days and cells were passaged when the confluence reached 80%. Cells at each passage (P0, P1 and P2) were collected with Trizol (Invitrogen) for RNA extraction or fixed with 4% paraformaldehyde (PFA) for toluidine blue staining.

*Toluidine blue staining*

The fixed cells were rinsed with PBS for 3 times and stained with 1% toluidine blue for 4h with shaking. The cells were then washed with water and observed. The images were captured using a Leica inverted microscope (Leica DM16000B) .

*Quantitative PCR*

RNA was extracted using Trizol (Invitrogen). RNA was reverse transcribed using the RevertAid First Strand cDNA Sythesis Kit (Fermentas) and qPCR was conducted with iQ SYBR Green Supermix (BioRad) using a ViiA 7™ Real-Time PCR Sytsem (Applied Biosystems); samples were normalized to glyceraldehyde- 3-phosphate dehydrogenase (Gapdh). Primers used in this study were the following: *5’-TGTCCTGTGCGACGACATAA-3’* and *5’-CCTTTGGTCCTGGTTTCCCA-3’* for *Col2a1*; *5’-GCTACGGAGACAAGGATGAGTTC-3’* and *5’-ATGGAGGGTGAGGTCTTTTACG-3’* for *Aggrecan*; *5’-GAATCCACTGGCGTCTTCAC-3’* and *5’-CGTTGCTGACAATCTTGAGAGA-3’* for *Gapdh*.

**Results**

Toluidine blue staining revealed production of GAGs gradually decreased from primary P0 to P1and P2 cells. Quantitative PCR also showed the expression of Col2a1 and aggrecan decreased with cell passaging.

Figure 1


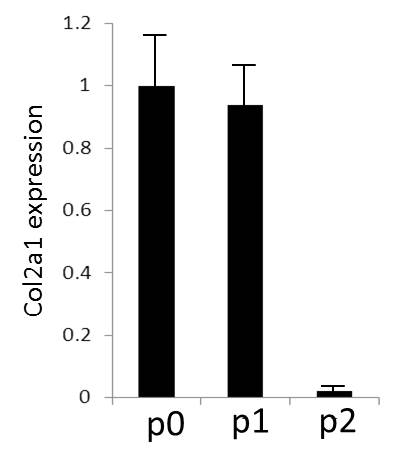

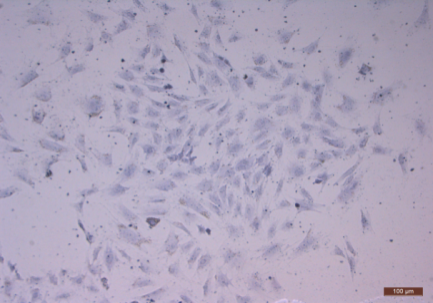


D

A


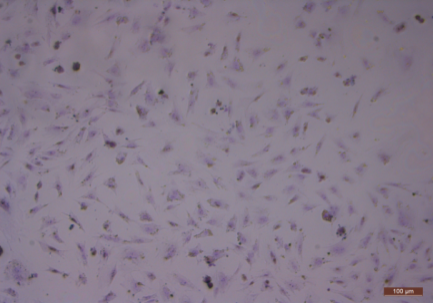


B


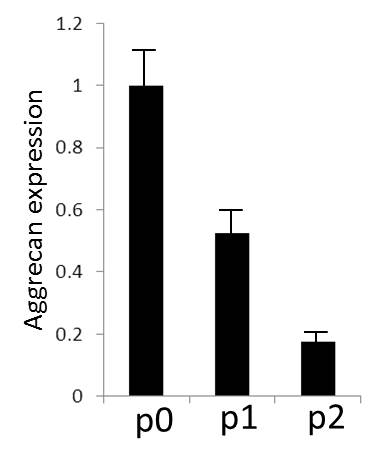


E

C


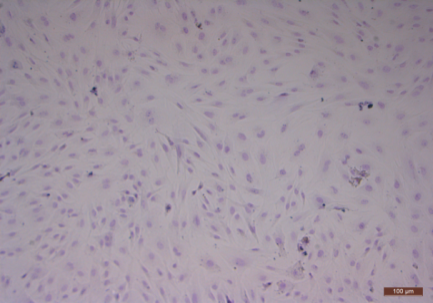


Figure1. Evaluation of GAGs, Col2a1 and aggrecan in primary and passaged rabbit chondrocytes. (A-C) Toluidine blue staining showed production of GAGs from cells declined following passaging from P0 (A), P1 (B) to P2 (C). Scale bar: 100μm. (D-E) Real-time PCR showed the expression of Col2a1 (D) and aggrecan (E) decreased with cell passaging.
